# Asystole During Nasopharyngeal Swab: Is COVID-19 to Blame?

**DOI:** 10.7759/cureus.15448

**Published:** 2021-06-04

**Authors:** Luai Madanat, Amal Khalife, Matthew Sims

**Affiliations:** 1 Internal Medicine, Beaumont Hospital, Royal Oak, USA; 2 Infectious Disease, Beaumont Hospital, Royal Oak, USA; 3 Foundational Medical Studies, Oakland University William Beaumont School of Medicine, Rochester, USA

**Keywords:** nasopharyngeal swab, sars-cov-2, asystole, trigeminocardiac reflex, vagal response, arrhythmias, covid 19

## Abstract

The use of nasopharyngeal (NP) swab sampling for the detection of various respiratory pathogens has been a standard procedure in medicine for many years. While this is a fairly common procedure, there has been a significant increase in utilization recently due to the SARS-CoV-2 pandemic. We describe a case of a 40-year-old SARS-CoV-2 positive patient with no prior cardiac history who developed asystole while an NP swab was being used to obtain a sample for a SARS-CoV-2 assay. Return of normal sinus rhythm was achieved with chest compressions alone. The incident was deemed to have been an exaggerated vagal response to intranasal stimulation; better known as the trigeminocardiac reflex. This is the first reported case describing asystole during use of an NP swab. This case occurred in a patient with no known cardiac disease and highlights the potential importance of the arrhythmogenic nature of COVID-19 that could potentiate the vagal response in susceptible individuals undergoing NP sampling.

## Introduction

Nasopharyngeal (NP) swab sampling has been widely utilized as a method to isolate viral and bacterial pathogens involved in upper and lower respiratory tract infections [1]. Amidst the recent severe acute respiratory syndrome coronavirus 2 (SARS-CoV-2) outbreak, a significant increase has been noted in the number of NP swabs performed throughout the world in an attempt to diagnose and contain the spread of this pandemic in both adults and children [2]. NP swabs are considered generally safe although more serious complications such as nasal abscesses, moderate to severe epistaxis, impacted swabs and cerebrospinal fluid leak have been reported in literature [3, 4].

Bradycardia induced by intranasal stimulation was described as early as the 1960s [5], however it has not been widely reported in literature thus far. Several reports were able to elicit the occurrence of apnea and bradycardia following nasal stimulation with ammonia in animal subjects [6-8]. The true risk of bradycardia during or immediately after use of an NP swab is unknown as in most cases it is done as an outpatient procedure where patients are not undergoing cardiac monitoring. If of short duration, even if the patient is symptomatic, the occurrence of bradycardia might never be noted. In our case, the patient was in the intensive care unit and on continuous cardiac monitoring and experienced an unusual episode of asystole lasting 25 seconds during NP swab sampling. This occurrence has not been described in literature following NP swab despite its widespread use. It is believed to be a result of vagally mediated nasocardiac reflex which is a variant of the well-known trigeminocardiac reflex (TCR).

## Case presentation

A 40-year-old Caucasian male with no prior medical history originally presented to an urgent care facility for shortness of breath and had tested positive for SARS-CoV-2. He was deemed to be stable and was sent home. The patient subsequently presented to a community hospital within our healthcare system the following week for continued difficulty breathing, persistent fevers and fatigue. Upon arrival, the patient was in acute hypoxemic respiratory failure with an oxygen saturation of 85% on room air. He was placed on 6L oxygen by nasal cannula and maintained adequate oxygen saturation.

Chest X-ray demonstrated prominent bibasilar patchy intestinal opacities in the lower lung fields. Labs were significant for WBC count of 15.2 bil/L and elevated inflammatory markers as follows: lactate dehydrogenase (LDH) 729 U/L, fibrinogen >1000 mg/dL, ferritin 1945 ng/mL, D-dimer 1376 ng/mL FEU, C-reactive protein (CRP) 438 mg/L, procalcitonin 0.39 (ref 0.00 - 0.25 ng/mL). Electrocardiogram (ECG) demonstrated sinus tachycardia with a rate of 114 bpm. The patient was started on remdesivir and dexamethasone 20 mg IV once daily. Despite this treatment, his respiratory status deteriorated with increasing oxygen requirements and was subsequently placed on a combination of high-flow nasal cannula and non-rebreather at 100% FIO2. He was started on vasopressors for worsening hypotension and IV azithromycin and cefepime due to concern for superimposed bacterial infection in the setting of elevated procalcitonin, low grade fevers and worsening hypoxemia and hypotension.

**Figure 1:**
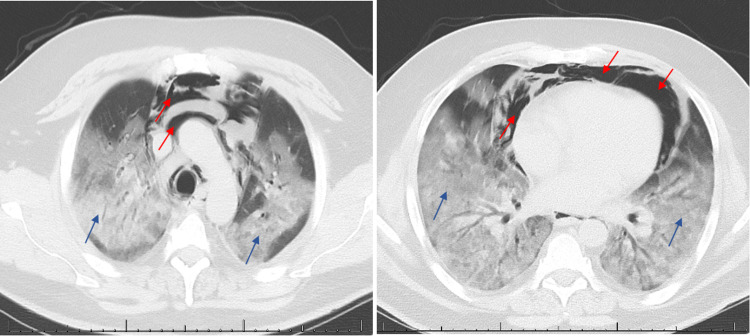
Chest CT scan demonstrating extensive pneumomediastinum (red arrows) with evidence of diffuse ground-glass opacities (blue arrows).

Despite medical therapy and supportive care, the patient’s condition continued to deteriorate. Chest CT scan demonstrated evidence of pneumomediastinum (Figure 1).

**Figure 2:**
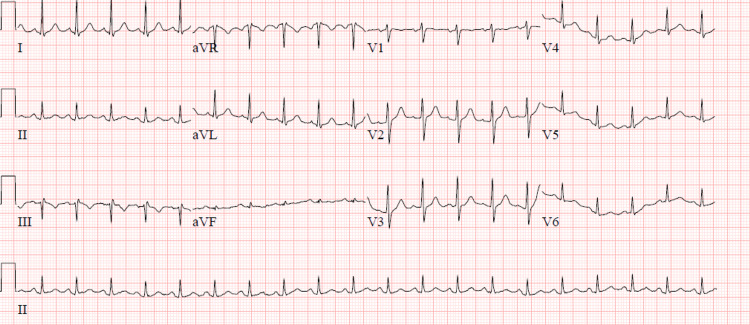
Patient’s baseline ECG upon arrival demonstrating sinus tachycardia with normal PR and QTc interval.

He was intubated and subsequently transferred to our facility for escalation of care and possible extracorporeal membrane oxygenation (ECMO). Upon arrival at our facility, the patient remained mechanically ventilated, and on vasopressors for refractory hypotension. Dexamethasone and remdesivir were continued for SARS-CoV-2 viral pneumonia, and IV antibiotics were continued for potential secondary bacterial pneumonia. The patient was given one dose of tocilizumab 800 mg IV. At this point a baseline EKG at our institution was obtained and demonstrated normal sinus rhythm (NSR) with both normal PR and QTc intervals (Figure 2).

On hospital day 2, a nasogastric tube (NG) was inserted and the patient was placed in prone position according to ARDSnet protocol. He was on propofol and fentanyl for sedation and cisatracurium for neuromuscular paralysis for optimization of mechanical ventilation. Vent setting were as follows: Mode PC/AC, FiO2 100%, respiratory rate (RR) 24, and positive end-expiratory pressure (PEEP) 15 cmH2O. COVID swab obtained on day of admission resulted positive and the patient was given convalescent plasma as part of a clinical trial (ABACCuS, NCT04432272). Over the following days, the patient’s respiratory status improved as evidenced by his decreasing ventilatory requirements, and paralytics were discontinued. By hospital day 6, the patient’s hemodynamic status had improved and was consequently weaned off IV vasopressors with completion of a course of IV cefepime, azithromycin and remdesivir.

**Figure 3:**
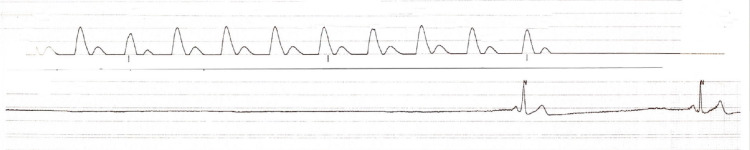
Telemetry strip during episode of asystole.

**Figure 4:**
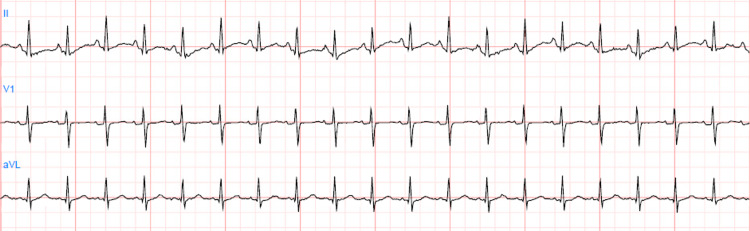
ECG following episode of asystole demonstrating return of normal baseline sinus rhythm.

On hospital day 7, labs were significant for WBC count of 12.8 bil/L, hemoglobin of 13 g/dL and normal procalcitonin of 0.04. Electrolytes were within normal limits as follows: Na 134, K 4.3, corrected Ca 8.88, PO4 3.4, Mg 2.2. The patient had a sinus rhythm of 84 bpm on telemetry. Ventilator settings were as follows: Mode PC/AC, FIO2 50%, RR 12, and PEEP 12 cmH2O. Sedation was maintained on fentanyl 150 mcg/hr and precedex 1 mcg/kg/hr. Enteral nutrition was continued via NG tube. The patient was not on any atrioventricular nodal blocking agents. Repeat nasal swab was performed on day 7 of admission per protocol for ABACCuS to determine if the previously given plasma had reduced the viral load of SARS-CoV-2. During the NP swab the patient had an immediate rhythm change from NSR to asystole (Figure 3). This persisted for 25 seconds during which pulse was non-palpable. Chest compressions were initiated per ACLS protocol; about 10 chest compressions were preformed after which return of spontaneous circulation (ROSC) was achieved with a palpable pulse and mean arterial pressure of 64 mmHg. The patient then reverted back into an organized sinus rhythm (Figure 4). This brief event was thought to be due to an exaggerated vagal response during the NP swab.

Ten days after presentation, the patient was successfully extubated and transferred out of the medical intensive care unit. His hospital course was complicated by positive blood and respiratory cultures for methicillin-sensitive Staphylococcus aureus. He was initially placed on IV vancomycin and subsequently switched to IV cefazolin based on susceptibility results. Follow-up echocardiogram demonstrated a left ventricular ejection fraction of 60%, no valvular vegetations or structural heart abnormalities were noted. Repeat COVID swab approximately three weeks following presentation was performed without issues and resulted negative. He was subsequently discharged home.

## Discussion

This patient had a prolonged complicated hospital stay as a result of SARS-CoV-2 infection. Despite receiving therapy for COVID-19 which was the standard of care at our institution at the time of his admission and maximal supportive care he was intubated for acute hypoxemic respiratory failure. On the day of his repeat nasal swab, the patient was noted to be in NSR and was not on vasopressors. Although the patient was given azithromycin in the days prior to the onset of asystole, there was no evidence of prolonged QT interval. Asystole demonstrated on the telemetry monitoring system developed as soon as the NP swab was inserted, and pulse was not palpable at that time. After several chest compressions, ROSC was achieved. This was thought to occur as a result of an exaggerated vagal response to nasal stimulation.

The TCR is a vagally mediated brainstem reflex defined as sudden alterations in blood pressure, heart rate and respiration manifested by apnea, hypotension, bradycardia and asystole as a result of stimulation of any of the branches of the trigeminal nerve along their course [9]. Three subtypes of TCR have been described in literature including peripheral, central and Gasserian ganglion type TCR. The nasocardiac, oculo-cardiac and the maxillo-mandibulo-cardiac reflex (V2-V3) are considered entities of the peripheral TCR [9]. There are several reports in the literature demonstrating vagally medicated cardiovascular effects including bradycardia after stimulation of the trigeminal nerve during ocular and maxillofacial surgeries [10, 11].

Baxandall and Thorn first described the nasocardiac reflex during which profound bradycardia was observed when a nasal speculum was introduced into the nares [12]. However, the incidence of asystole after intranasal stimulation remains scarce with only two cases published to date. Bailey demonstrated the occurrence of sinus arrest following introduction of a temperature probe into the nasal cavity. The heart rate was immediately restored to normal upon removal of the prob. However, it is worth mentioning that in that report, sinus arrest occurred following administration of cardio-depressive medication such as thiopental, droperidol and vecuronium which might have contributed to asystole [13]. A recent report highlighted the incidence of asystole with sinus arrest during the administration of local anesthetic in the nasal cavity. At the time of nasal puncture, the patient developed sudden asystole lasting 10 seconds followed by sinus bradycardia. Repeat nasal puncture led to sinus bradycardia at 30 bpm [14].

Cardiovascular manifestations of COVID-19 infection have been recognized as an important complication among hospitalized patients. Arrhythmias have been frequently described in patients with SARS-CoV-2 pneumonia [15, 16]. In a recent worldwide survey of COVID-19 associated arrhythmias including 4526 patients, 827 (18.3%) were reported to have some sort of arrhythmia [16]. Of those, 81.8% developed atrial arrhythmias, 20.7% developed ventricular arrhythmias and 22.6% developed bradyarrhythmias including sinus bradycardia, AV block and sinus pause. Sinus bradycardia is one of the common arrhythmias observed in COVID-19 with reports demonstrating persistence of bradycardia for up to two weeks [17]. Previous reports have postulated that the mechanisms behind arrhythmia in COVID-19 may include cardiac damage from metabolic derangements, hypoxia, inflammatory or neurohormonal stress, and myocarditis [18, 19]. While cardiac manifestations are now a recognized sequela of COVID-19 infection, mechanisms behind arrhythmogenic complications remain less understood and studied in literature.

Management of peripheral TCR is based on anecdotal data with no clear treatment algorithms established thus far. Cessation of the offending stimulus is the first and most important step in the management of TCR and often leads to successful restoration of baseline hemodynamic parameters [20]. In our case, the patient went into asystole immediately following introduction of the swab into the nasopharynx. The swab was instantly removed and chest compressions initiated. Pulse was regained and NSR established following several chest compression. Sinus rhythm was maintained with adequate blood pressure and the patient did not have further episodes of bradycardia or asystole throughout his hospital stay. We speculate that the arrhythmogenic effects of COVID-19 played a significant role in the development of asystole in our patient due to the rarity of this occurrence following NP swabs and the lack of other identifiable factors that may have contributed to asystole.

## Conclusions

This case highlights the importance of recognizing peripheral variants of TCR. It is the first case reported to document asystole during a nasopharyngeal swab. While this is the first case to report such a phenomenon, it is possible that the occurrence of an exaggerated vagal response is often missed as the majority of NP swabs are performed in patients who are not on continuous cardiac monitoring. Further research is needed to shed light on potential risks and aggravating factors that might contribute to occurrence of peripheral TCR. Treatment algorithms must be developed to help manage cases in which removal of the noxious stimulus does not result in restoration of normal hemodynamics.
